# Engineered chitosan for improved 3D tissue growth through Paxillin-FAK-ERK activation

**DOI:** 10.1093/rb/rbz034

**Published:** 2019-09-30

**Authors:** Md Abdul Kafi, Khudishta Aktar, Mitsugu Todo, Ravinder Dahiya

**Affiliations:** 1 BEST Group, School of Engineering, University of Glasgow, Glasgow G12 8QQ, UK; 2 Department of Microbiology and Hygiene, Bangladesh Agricultural University, Mymensingh 2202, Bangladesh; 3 Research Institute for Applied Mechanics, Kyushu University, Kasuga, Fukuoka, Japan

**Keywords:** adhesion signaling, chitosan, hMSC, porous scaffold, proliferation, Cys-RGD, tissue engineering

## Abstract

Scaffold engineering has attracted significant attention for three-dimensional (3D) growth, proliferation and differentiation of stem cells *in vitro*. Currently available scaffolds suffer from issues such as poor ability for cell adhesion, migration and proliferation. This paper addresses these issues with 3D porous chitosan scaffold, fabricated and functionalized with cysteine-terminated Arg-Gly-Asp (Cys-RGD) tri-peptide on their walls. The study reveals that the compressive moduli of the scaffold is independent to RGD functionalization but shows dependence on the applied freezing temperature (TM) during the fabrication process. The low freezing TM (−80°C) produces scaffold with high compressive moduli (14.64 ± 1.38 kPa) and high TM (−30°C) produces scaffold with low compressive moduli (5.6 ± 0.38 kPa). The Cys-RGD functionalized scaffolds lead to significant improvements in adhesion (150%) and proliferation (300%) of human mesenchymal stem cell (hMSC). The RGD-integrin coupling activates the focal adhesion signaling (Paxillin-FAK-ERK) pathways, as confirmed by the expression of p-Paxillin, p-FAK and p-ERK protein, and results in the observed improvement of cell adhesion and proliferation. The proliferation of hMSC on RGD functionalized surface was evaluated with scanning electron microscopy imaging and distribution though pore was confirmed by histochemistry of transversely sectioned scaffold. The hMSC adhesion and proliferation in scaffold with high compressive moduli showed a constant enhancement (with a slope value 9.97) of compressive strength throughout the experimental period of 28 days. The improved cell adhesion and proliferation with RGD functionalized chitosan scaffold, together with their mechanical stability, will enable new interesting avenues for 3D cell growth and differentiation in numerous applications including regenerative tissue implants.

## Introduction

Porous scaffold has been extensively used in recent years in tissue engineering, regenerative medicine, replacement therapies, implants and neural interfaces, etc. [1, 2]. Scaffolds with adequate cell adhesion ability and homogenous porous structure, allowing firm cell adhesion, copious supply of nutrients [3] and uniform proliferation, are critical for 3D growth and proliferation of cells in above applications. Ideally, the scaffold material should also have tunable mechanical properties, for example, to match with the mechanical stiffness of target tissues [4]. Wide varieties of synthetic materials and biomaterials such as polymer, bio ceramics, chitosan, collagen, poly-l-lysine, laminine, etc. have been reported for porous scaffolds [5–7]. However, scaffolds from majority of these materials offer poor cell adhesion and migration [8, 9], which, as said above, are needed for 3D growth and proliferation. In this regard, chitosan is interesting as its monomer units [(C_12_H_24_N_2_O_9_)_n_] with numerous hydroxyl (–OH) and amino groups (–NH2) offers excellent cross-linking features needed to attain stability against ordinary solvent [10]. Another distinct feature of chitosan is that its mechanical properties are tunable, as demonstrated later in this paper. Moreover, the excellent features of chitosan such as biological renewability, biodegradability, biocompatibility and non-allergic and antimicrobial activities [11–13] as evident from its use in several medical applications such as wound dressing, space filling and drug delivery are considered as other attributes to their implant ability [14]. With adhesion molecule (AM) functionalization, these monomer units also can lead to improved cell adhesion and proliferation. AM is essential for focal adhesion (FA), spreading and proliferation of mammalian cells [15, 16]. In fact, the initiation electrostatic interaction activates cellular integrin receptor for the formation of biomechanical linkage with AM available on the surfaces followed by the activation of FA. The FA depends on the spatial nanoscale arrangement aligning with the arrangement of the integrin receptor on the cell membrane [15, 16]. Therefore, nanoscale dotted arrangement of AM is desirable instead of their continuous layers like arrangement. However, majority of previous research on AM functionalization in 3D scaffold has not been explored this issue [17–19]. They functionalized AMs on chitosan molecules during scaffold formation process without considering their arrangement and distribution throughout the scaffold and thus adhesion, proliferation and migration of cells were inadequate for the regeneration of 3D tissue implant (Table 1). Here we developed porous 3D chitosan scaffold functionalized with homogenously distributed nanodot AM molecules for alignment with the integrin receptors and thus developed an advanced platform for the 3D tissues growth. In fact, this type of nanoscale arrangement of AM peptide has not been explored in any 3D scaffold made from the wide range of materials mentioned above. Herein, we present advanced porous chitosan 3D scaffolds showing enhanced cell growth and proliferation capability and temperature (TM)-dependent tunability of mechanical stiffness. The scaffolds was developed using phase separation and lyophilization method and functionalized with AMs residing in the ECM proteins, which are rich in repeated sequences of Arg-Gly-Asp (RGD) tripeptides [20]. Cysteine-terminated RGD have been widely used in artificial surface modification for mimicking cell functions [16] such as adhesion, spreading, proliferation, differentiation and gene expression [16, 21]. However, the exact molecular mechanism of such adhesion dynamics in AM functionalized 3D scaffold has not explored thus far (Table 1). Filling this gap, this research also demonstrates the molecular mechanism behind observed improvement of FA by monitoring of associated proteins expressions in cell grown scaffold. The RGD peptide involves cell surface recognition and strong cell-substrate adhesion, which lead to the activation of FA signaling pathways [22–24]. These FAs signaling pathway guide the cellular spreading and proliferation [25]. More specifically, the ability for strong adhesion of RGD functionalized surfaces activates the transmembrane protein (integrin), which initiates FA signaling pathway through a series of protein activations such as tallin-paxillin-Src-FAK for cytoskeletal assembly and spreading, and Src-FAK-ERK for spreading and migration [23, 25, 26]. This said, the introduction of RGD functionalization is challenging because additional cysteine residues are needed at the end of peptide to facilitate their coupling (–S: S–) with thiolated material surfaces, as we reported in the past [16, 20, 27]. An addition of thiol group (–SH) chemical on chitosan backbone, prior to the introduction of cysteine-terminated RGD molecules, can help to solve this issue [28] and this has been successfully explored in this paper.

**Table 1:** Comparison between AM functionalization strategies on chitosan scaffold for enhancing cell adhesion and proliferation

Types of AMs	Functionalization strategy	Uniformity of RGD	Cell line	100% confluences	Molecular mechanism	Ref.
RGD-Ser	Imide bond formation	Non	ROS	Not achieved	Not studied	[17]
GRGDGY	Schiff base formation	Non	HDF	Not achieved	Not studied	[18]
RGD	Covalent linkage	Non	ATDC5	Not studied	Not studied	[29]
GRGDS	Grafted on CM-TM-Chitosan	Non	HDF	Not studied	Not studied	[19]
GRGDSPGYG	Azido group formation	Non	MSCs	Not achieved	Not studied	[30]
Cys-RGD	Disulfide bonding (–S: S–)	Uniform	hMSC	Achieved within three WKs	Paxillin-FAK-ERK activated adhesion	This work

ROS, rat osteosarcoma; HDF, human dermal fibroblast; ATDC5, Murine chondrogenic cell.

## Materials and methods

### Materials

All reagents and chemicals were of puriss grade quality and were used as received. Chitosan (degree of de-acetylation 98%) was obtained from Dainichiseika Color & Chemical Mfg. Co. Ltd. (Japan). Cysteine-terminated RGD peptide was obtained from Bachem AG Sigma (USA). Cell counting kit was purchased from Dojindo. Materials for cell culture including α-minimum essential medium eagle with l-glutamine and phenol red (MEMα) and ethanol were from Wako Pure Chemical Industries, Ltd.; phosphate buffered saline, trypsin and fetal bovine serum (FBS) were from Gibco, Trepan blue solution (4%) and penicillin-streptomycin (Pen-Strep) were from Sigma. Anti-FAK, anti-phospho-FAK (Tyr-397), anti-ERK1/2 and anti-p-ERK antibodies were obtained from Cell Signaling (MA, USA). Anti-p-Paxillin (Y118) antibody was obtained from Abcam (Cambridge, UK). Anti-actin antibodies were obtained from Sigma (Missouri, USA).

### Porous chitosan scaffold fabrication

**Figure 1:**
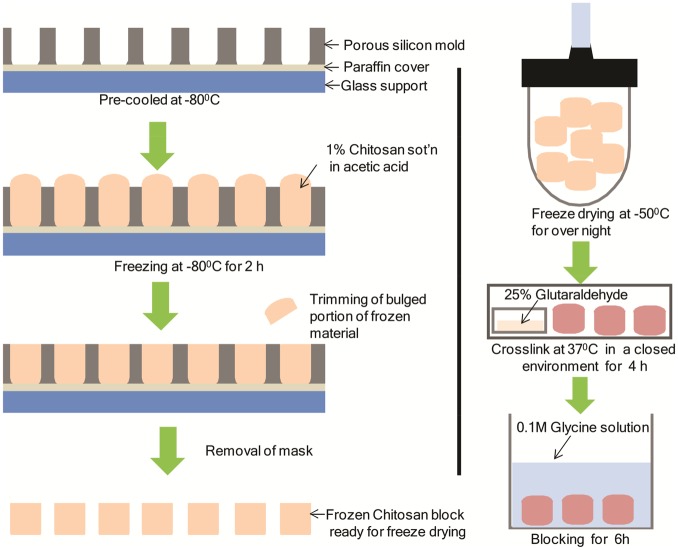
Schematics representation of systematic fabrication of porous chitosan scaffold using phase separation and lyophilization method. Two different freezing TM (−80°C and −30°C phase) were applied separately to produce scaffolds of two TM groups

The chitosan solution (1 wt%) was prepared in a solution of 2 M acetic acid and homogenous solution was achieved by stirring with a magnetic stirrer. The chitosan solution was preserved at a TM of 4°C for future use. The chitosan solution was poured on a pre-cool silicon mold with a dimension of 1 cm × 1 cm and freezes at a relatively high freezing TM −30°C and −80°C for 2 h to promote the formation of larger sized ice crystals. The frozen suspension was subsequently lyophilized for 24 h to obtained porous scaffold with a dimension of 1 cm × 1 cm. To enhance structural stability of chitosan scaffolds, lyophilized samples were cross-linked with 25% glutaraldehyde solution (Wako Pure Chemical Co. Ltd) in an oven at a TM of 37°C for 24 h. These cross-linked scaffolds were immersed in a 0.1 M glycine solution for 4 h at room TM for blocking free aldehyde groups. Following the cross-linking, scaffolds were washed with distilled water (six times) and subsequently re-frozen re-lyophilized for obtaining stable porous chitosan scaffold. The detail fabrication steps are illustrated in Fig. 1.

### RGD functionalization of chitosan scaffold

For RGD functionalization, chitosan scaffold was thiolated with 4-thiobutyrolactone (Aldrich Co., USA) as described elsewhere [28]. Briefly, each lyophilized chitosan scaffold was placed in each well of a 24-well plate and 400 µl of deionization water was added. An aqueous solution of imidazole (Aldrich Co., USA) (50 µl in 400 µl) was added, followed by dropwise addition of 4-thiobutyrolactone (300 µl) on a constant plate shaker. After 12 h, the scaffold was taken out and placed in a fresh 24-well plate and washed with 1 wt% sodium dodecyl sulfate (SDS) and a 1 N NaCl aqueous solution to remove adsorbing reagents and finally with distilled water. After removing unreacted reagents and imidazole, the thiolated chitosan scaffold was dried in a vacuum. Subsequently, RGD peptide terminated with cysteine amino acid was deposited on thiolated chitosan scaffold by submersing in the peptide solutions (0.1 mg/ml in PBS) for 12 h at 4°C. Thus, the thiolated chitosan scaffold was functionalized with Cys-RGD, where thiol groups are covalently linked (–s: s–) with each other to organize in a nanodot arrangement (Fig. 3a and c).

### Mechanical testing

The mechanical testing of the cell-cultured scaffold as well as the pristine scaffold was performed throughout the experimental period to confirm their mechanical stability and practical applicability in tissue engineering. For this, specimen’s dimensions of L (mm) in length, W (mm) in width and *H* (mm) in height were measured prior to the test. Compression tests were performed by using Shimadzu Compact Tabletop Testing Machine EZTest (EZ-S Series) equipped with 500 N load cell and a crosshead speed of 1 mm/min. Force F (N) and displacement Δ*H* (mm) were determined and then stress σ (kPa) (Equation 1.1) and strain ε (Equation 1.2) were calculated. Strength was acquired from the stress values of the stress-strain curve just before the cracking of specimen. For each condition, five samples were tested and average value was used.

(1.1) $$\text{Stress }\rho =\frac{F}{L\times W},$$

(1.2) $$\text{Strain }\varepsilon =\frac{\Delta H}{H}.$$

### Human mesenchymal stem cell seeding and culture

Human mesenchymal stem cell (hMSC) (UE6E7T-3, Riken Bioresource Center) was cultured and maintained in standard growth medium (MEMα) supplemented with FBS (10%) and Pen-Strep (1%). The cells were maintained in an incubator providing standard culture conditions at 37°C in an atmosphere of 5% CO_2_ and 70% humidity and feed twice per week and subculture when 90% confluences attained. All the experiments were performed under identical condition with cells from 4th passage. Prior to cell seeding, porous chitosan scaffold was placed in 48-well plate at an aseptic condition and allow UV treatment to confirm microbiological sterility. Then, scaffold with improved mechanical stability (from −80°C) was subjected to hMSC cell seeding at a concentration of 1.0 × 10^5^ cells/scaffold and placed under cell culture environment throughout the experimental period to determine cellular adhesion and proliferation efficiency.

### hMSC adhesion and proliferation assay

The cell-scaffold adhesion strength was measured by using centrifugation adhesion assay to obtain the fraction of adherent cells after centrifugation at a defined force as a metric of comparison. For that, thiolated porous chitosan materials were immersed in 0.1% RGD solution and kept overnight at 37°C. Then RGD-coated scaffolds were moved to a fresh 24-well plate and washed with culture medium and subsequently seeded with 1.0 × 10^5^ cell for 6, 12, 18 and 24 h. The scaffold without RGD was maintained in parallel throughout the experiment as a control, where loosely bound or non-bound cell were washed away from the scaffold due to centrifugal force leaving the attached cell only. At the end, scaffold was taken out and placed in a fresh plate for cell counting using the kit from Dojindo. Prior to cell counting, the cultured scaffold was washed twice with PBS to remove culture medium. Then, cells were incubated in 500 µl PBS with 50 µl reagent in a humidified atmosphere at 37°C and 5% CO2 for 2 h. Finally, 110 µl of solution was transferred to 96-well plates and OD value measured by a plate reader (Perkin Elmer Arvo X2) at 37°C with wavelength of 450 nm. For cell proliferation assay, each scaffold was seeded with 1 × 10^5^ cell and maintained for 1, 7, 14, 21 and 28-day under cell culture environment. Cell counting was performed at 7-day interval for monitoring the cell numbers throughout the experiment.

### Western blot analysis

RGD-integrin coupling activated focal adhesion kinase (FAK) signaling was confirmed by western blot analysis of p-Paxillin, p-FAK and p-ERK proteins [25, 26] in cells grown on scaffold. The hMSC proliferated scaffolds (*n* = 3) were washed with PBS and frozen in liquid nitrogen at each time point and stored at −80°C. Each of the frozen scaffolds was crashed using sterilized pastel and mortar in lysis buffer 50 mM Tris (pH 7.7), 150 mM NaCl, 0.5% NP-40, 10% Glycerol, 1 mM DTT, Protease inhibitor and DDW. The supernatant after centrifugation was recovered and protein content were quantified by the Bradford assay (Bio red Laboratories). Total protein separated by electrophoresis on 10% and 12% SDS-polyacrylamide gels on size of target protein was investigated. The proteins were electroblotted on to nitrocellulose membranes, probed with primary antibodies overnight and re-tripped in secondary antibody after washing with PBS.

### Scanning electron microscopy

Imaging and histochemical characterization of hMSC culture scaffolds have performed every week for 4 weeks to confirm cell proliferation and distribution throughout the scaffold. A scanning electron microscope (SEM, Hitachi, Ltd. S-4100) was used to determine pore structures of the fabricated scaffolds. Prior to SEM imaging, samples was sputter coated with a thin (3–5 nm) layer of gold (Au) for observation at an accelerated voltage of 10 kV and 10 mA current, while working distance was adjusted for various levels of magnification. The longitudinal section, top and the bottom extremes of the scaffold were examined to identify any differences in pore size across the sample. In case of cell-cultured scaffolds, samples were fixed in 4% paraformaldehyde and dehydrate prior Au sputtering. The cell fixed scaffolds were achieved by slow dehydration process using gradually increased concentrations of ethanol and t-butyl alcohol solutions (Wako Pure Chemical Co. Ltd).

### Hematoxylin and eosin staining of hMSC-cultured chitosan scaffold

For hematoxylin and eosin (H&E) staining hMSC-cultured chitosan scaffolds were fixed in 4% paraformaldehyde solution and slowly dehydrated using graded ethanol (10–70%) followed by embedded in paraffin for microtome section at 5 µm thickness. Sections were stained with H&E as per protocol describes elsewhere [31]. Stained sections were examined using Nicon Eclipes 80i Microscope.

### Statistical analysis

Data were compared using analysis of variance (ANOVA) followed by Bonferroni posttests. Statistical analyses were performed at 95% confidence level using GraphPad Prism version 5.0. All values are expressed as mean ± standard deviation from three to five individual samples.

## Results and discussion

### Fabrication and characterization porous chitosan scaffold

**Figure 2:**
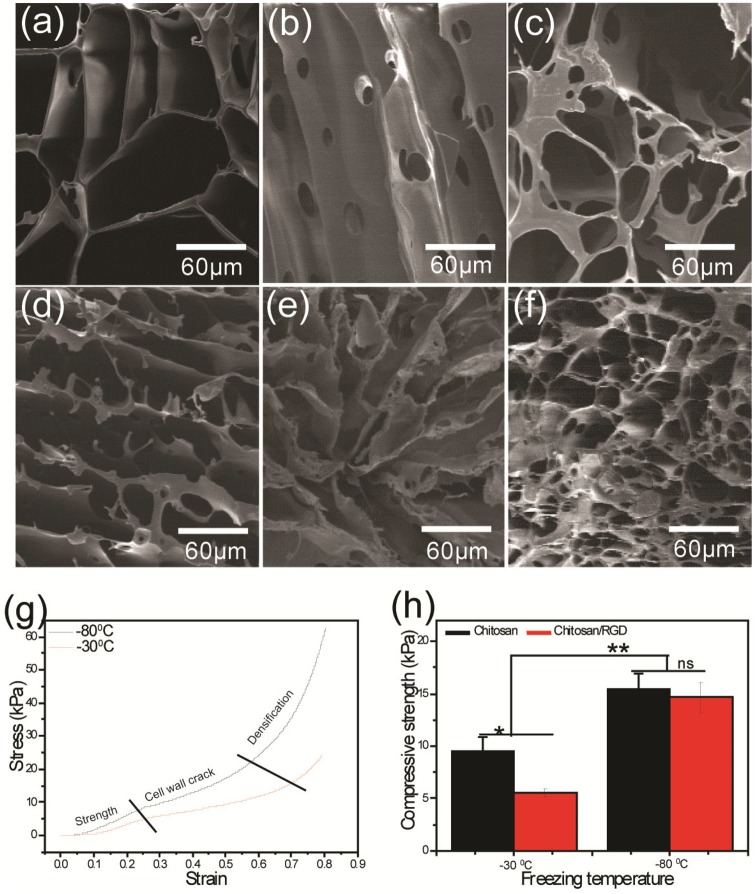
SEM images of the chitosan scaffold fabricated at −80°C (**a–c**) and −30°C (**d–f**), where a and d were taken from top, b and e from longitudinal section and c and f from bottom. Scale bar for all images 60 μm. The images were obtained using a field emission SEM (Hitachi, Ltd. S-4100) operating at an acceleration voltage of 10 kV and current 10 mA. All samples were coated with a conductive 3–5 nm sputtered gold layer prior to analysis. (**g**, **h**) Mechanical characterization of the fabricated scaffolds. (g) Stress-strain curve obtained from the scaffolds fabricated at two different freezing TMs. (h) Compressive strength of the fabricated scaffolds before and after RGD modification for the both TM group. All values are expressed as mean ± standard deviation from three individual samples. Asterisk indicates significant difference (**P < 0.01 and *P < 0.05)

Tissue engineering encompasses development of functional substitutes for damaged tissues and organs as alternative to auto or allograph. Typically, tissue engineering involves seeding cells in 3D porous biomaterial scaffold that provide mechanical support and instructive cues for the developing engineered tissue. Considering biocompatibility, biodegradability and self-renewability, chitosan is widely used in tissue engineering [11–13]. Herein, chitosan scaffold was formed by freezing chitosan solution in a pre-cooled silicon mold and subsequently lyophilizing the frozen structure for evaporating solubilizing agents resulting pores encircled with chitosan materials (Fig. 1). The TM-dependent variation in pore size was achieved when the suspension was subjected to two different freezing TM (−30°C and −80°C), resulting in scaffolds with two different porous structures (Fig. 2a–f). Generally, during freezing process closely packed chitosan molecules surround ice crystals [32]. During lyophilization, the ice crystal evaporates out and leaving micro porous structure encircled with solid chitosan materials, which serves as a nucleus of the scaffold [33]. Therefore, different TM result in microstructure ice crystal with varying size and this obviously produces scaffolds with different pore sizes. Fig. 2a–c shows SEM images of the top, longitudinal section and bottom surface of fabricated scaffolds at −80°C and SEM images from corresponding areas (mentioned above) of scaffold fabricated at −30°C are shown in Fig. 2d–f. TM-dependent morphologies of two scaffolds are revealed in the SEM images. Relatively stable scaffold was obtained when −80°C TM was applied during freezing process, whereas comparatively unstable scaffold was achieved with −30°C freezing TM. However, both TM groups had interconnectivity among neighboring pores, which is essential for the transport nutrients and growth factors during *in vitro* cell culture and maintenance [3].

Scaffolds from both TM groups were subjected to mechanical testing to determine their structural stability. The stress-stain curve obtained from scaffold of both TM groups showed similar trend, but variation in yield strength, cracking and densifications as illustrated in Fig. 2g. The scaffold fabricated at −80°C freezing TM showed stronger mechanical behavior than those fabricated at −30°C. The higher compressive strength was observed from scaffold fabricated at −80°C compared with the one at −30°C and this variation was observed from both RGD functionalized and pristine scaffold (Fig. 2h). However, a significantly decreased compressive strength was observed from RGD functionalized chitosan scaffold fabrication at −30°C (Fig. 2h). This reduction of compressive strength reflects low mechanical stability of scaffolds, which was prone to degrade during several washing steps followed by chemical modifications during RGD functionalization process [11]. However, compressive strength of scaffold developed at −80°C was significantly higher than their counterpart in high freezing TM. In addition, uniformity and interconnectivity of the pores structures are well distinct in scaffold from −80°C. Based on this structural stability and uniformity of porous structure, the scaffold fabricated at −80°C was chosen for hMSC culture and proliferation experiment.

### RGD functionalization of chitosan scaffold

**Figure 3:**
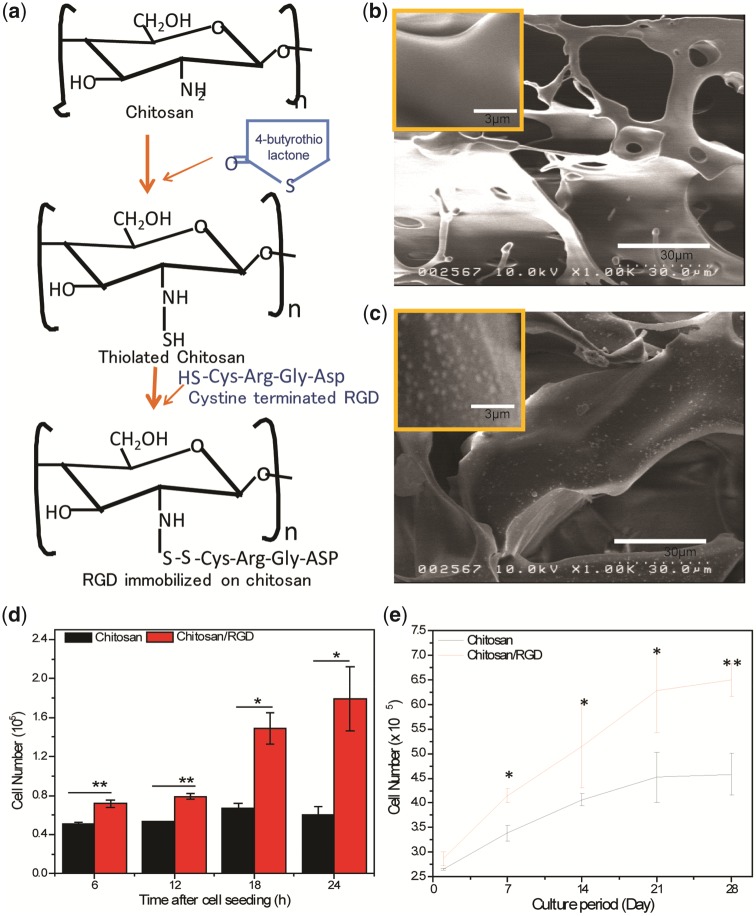
(**a**) Schematic elastration of RGD functionalization of chitosan. SEM images of prestine chitosan scaffold (**b**) and RGD functionalized chitosan surface (**c**). Scale bar for main images 30 μm and scale bar for inserted images 3 μm. (**d**) Evaluation of adhesion strength of hMSC cells on RGD functionalized chitosan scaffold and pristine chitosan scaffold after 6, 12, 18 and 24 h post seeding. (**e**) Proliferation of hMSC cells on RGD functionalized chitosan scaffold as well as pristine chitosan scaffold at 1, 7, 14, 21 and 28-day post seeding. All values are expressed as mean ± standard deviation from five individual samples. Asterisk indicates significant difference (**P < 0.01and *P < 0.05) as compared with RGD non-functionalized samples

The scaffold’s microenvironments that can attach and proliferate hMSC need to be organized into tissues with structural and physiological features resembling actual structures in the body [24, 34]. For the effective cell adhesion and proliferation, artificial surface requires modification with RGD tri-peptide sequences which generally resides as cell AMs in extracellular matrix [15, 16]. In the *in vivo* condition, extracellular matrix and its components fibers, adhesion proteins, proteoglycans, etc. provide wealth of information, which can regulate attachment, spreading, proliferation, migration and differentiation [35].To mimic these physiologic features, chitosan scaffold was functionalized with RGD peptide sequence having affinity to integrin receptor of cell surface [15, 16]. The RGD immobilization requires modification with an additional cysteine residue containing thiol (–SH) group, which can be coupled with thiolated surface [20].This cysteine-terminated RGD peptide sequence has already been proven successful for immobilization on Au surface using thiol-gold coupling method [27]. Herein, we have introduced –SH group onto chitosan scaffold for establishing a reaction between –SH groups of thiolated chitosan and cysteine-terminated RGD peptide. To achieve this activation, chitosan scaffold was reacted with imidazole and thiobutyrolactone simultaneously prior to RGD introduction [28]. The details process of chemical modification is illustrated in Fig. 3a. The RGD-modified chitosan scaffold was confirmed by analysing SEM images obtained from RGD functionalized as well as pristine chitosan surface. The clean surface without any deposition of materials was observed on pristine chitosan surface (Fig. 3b). Whereas a remarkably rough surface with nanoscale dots were revealed on the RGD fictionalized surface (Fig. 3c). This topological alteration was due to the assembly of nanoscale Cys-RGD molecules on the thiolated chitosan surface. Several previous works have reported self-assembly of RGD nanodots on the thiolated surface for enhancing cell adhesion ability of artificial surfaces [15, 16]. The nanoscale RGD-modified surface enhances early surface recognition stage of cell adhesion process and confers firm adhesion by coupling with cellular transmembrane protein (integrin) [22, 23]. This transmembrane protein also conveys topographical stimulation to several intracellular pathways to influence the cellular spreading, proliferation and differentiation [22, 23]. Therefore, the RGD functionalized chitosan scaffold can be a suitable platform for cell adhesion, spreading, proliferation and differentiation. Additionally, the interconnectivity of the pores structures of the fabricated chitosan scaffold will favor the cell functions by the easy access of nutrient and growth factors [36].

### hMSC adhesion and proliferation on fabricated porous scaffold

The cell adhesion assay was performed to assess the performance of RGD-modified chitosan scaffold over pristine chitosan scaffold. The adhesion strength assay was carried out by applying centrifugal forces on hMSC seeded RGD functionalized chitosan scaffold as well as pristine chitosan scaffold after which non-bound and weakly bond cells were washed off and the remaining cells were counted [37]. The remaining cell densities in the scaffolds at 6, 12, 18 and 24 h post seeding are plotted in Fig. 3d. The results reveal that RGD functionalized chitosan induced strong cell adhesion that withstands centrifugal force, resulting in high cell densities. Besides, low cell density was observed for scaffold without RGD indicating that the cells remained unattached or weakly attached to the pristine chitosan scaffold. Numbers of cell attached with the scaffold increased with adhesion times for the RGD functionalized scaffolds and pristine chitosan scaffolds. The significant enhancement (30%) of cell adhesion was noticed in RGD functionalized scaffold compared with pristine chitosan scaffold, because RGD molecule formed covalently linked with the thiolated of chitosan molecules and established stronger interaction with integrin receptors present on the cell surface [20]. This RGD-mediated enhancement of cell adhesion strength could have positive effects on FAK signaling pathway for enhanced spreading, migration as well as proliferation of hMSC [20, 22, 23, 38].

This RGD-integrin coupling enhanced proliferation behavior of hMSC was investigated on RGD functionalized scaffold comparing with pristine chitosan scaffolds. For that, cell seeded RGD functionalized and pristine scaffold were maintained in growth medium providing identical culture condition as mentioned earlier for 28 days. Representative sample from both groups was subjected to cell count every week and results are illustrated in Fig. 3e. The higher proliferation rate was recorded throughout the experimental period when RGD functionalized scaffold was employed. However, similar trend, but slower growth rates, was observed from pristine scaffold. In both cases, hMSC tends to grow exponentially up to 14 days and then slow growth rate was observed up to 21 days. Besides, decreased rate or no growth was recorded at 28th day when the maximum cell attaching surfaces are occupied. This feature of growth curve is obvious in *in vitro* condition due to the unavailability space where cell-cell contact inhibition growth might have occurred [39]. It is assumed that most of the scaffold surface was occupied within 21 days of culture. Usually, in *in vivo* condition macrophages play an important role in creating the spaces for new cell division by engulfing the decayed cell of tissue [33]. Hence, this limitation could be overcome in implanted engineered scaffold by host enzymatic and macrophage activity.

### RGD-integrin activated FA pathway

**Figure 4:**
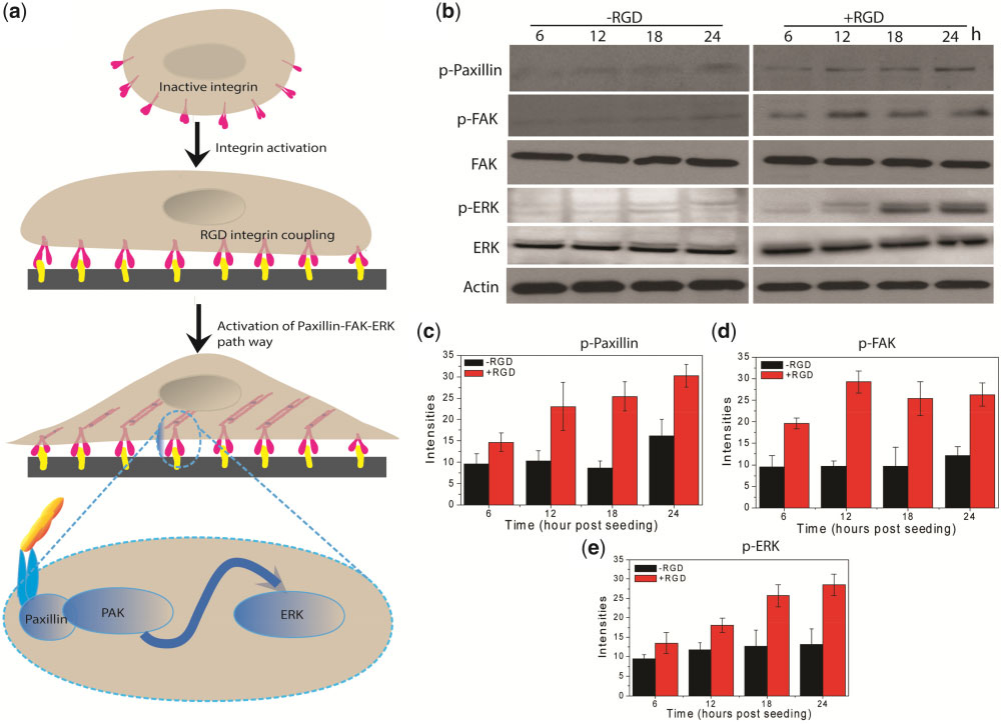
RGD-induced activations of FA pathway. (**a**) Schematic illustration of molecular mechanism behind the RGD-integrin coupling activated FA mechanism. (**b**) Western blots of proteins involved with FA-mediated cellular adhesion and proliferation. (**c–e**) Band intensities of p-paxillin, p-FAK and p-ERK blots, respectively. All values are expressed as mean ± standard deviation from three individual blots. Asterisk indicates significant difference (**P < 0.01and *P < 0.05) as compared with RGD non-functionalized samples

Cell-substrate adhesion is an important step for *in vitro* tissue engineering, which involves series of events such as surface recognition, early attachment stage, membrane adhesion and cell spreading [40]. The surface recognition activity is a weak nonspecific interaction between pericellular coat and substrate that happen when cell-substrate gap is <1 µm [40]. When cell-substrate gap reaches <100 nm, the membrane recognition proteins (as integrin) form strong bonding with the target RGD molecules on the surfaces, which is known as early attachment stage (Fig. 4a). Once cell attached firmly through RGD-integrin coupling, membrane become closer and cell-substrate gap become <10 nm, the transmembrane protein convey extracellular signal to activate FA signaling pathway (Paxillin-FAK-ARK) for actin cytoskeletal growth and organizations for spreading, migration and proliferation (Fig. 4a bottom inset) [25]. These cascades of events involved in actin cytoskeletal stretching and cellular spreading, which ultimately ends with cell divisions [16]. The RGD-integrin coupling assisted activation of FA pathways was confirmed by the western blotting analysis of p-Paxillin, p-FAK and p-ERK proteins [23]. The western blot analysis reveals significant differences in expressions of p-Paxillin, p-FAK and p-ERK in cells grown on the RGD functionalized surface compared with nonfunctionalized pristine surface (Fig. 4b). Fig. 4c shows significant enhancement in the expression of p-Paxillin in cell cultured on RGD functionalized surface compared with that of pristine surface. This enhancement continued with seeding period and maximum enhancement was achieved on day 24 post seeding. The p-FAK also showed similar enhancement in expressions, but the maximum enhancement was achieved at day 12 post seeding indicating maximum saturations in FAs by that period (Fig. 4d). A time-dependent increase in p-ERK expressions were reported throughout experiment, indicating the increased proliferations of the cells on the RGD functionalized surface (Fig. 4e). It is well known that paxillin protein activation is dependent on integrin-mediated transduction of extracellular signals from RGD-integrin coupling, which initiates the FA pathway by activation of FAK and ERK [25]. The increased expressions of p-paxillin, p-FAK and p-ERK levels in the cells from the RGD functionalized scaffold clearly indicates that the RGD-integrin activate the FA signaling pathway [22, 23].

### SEM morphology of cell-cultured RGD functionalized surface

**Figure 5:**
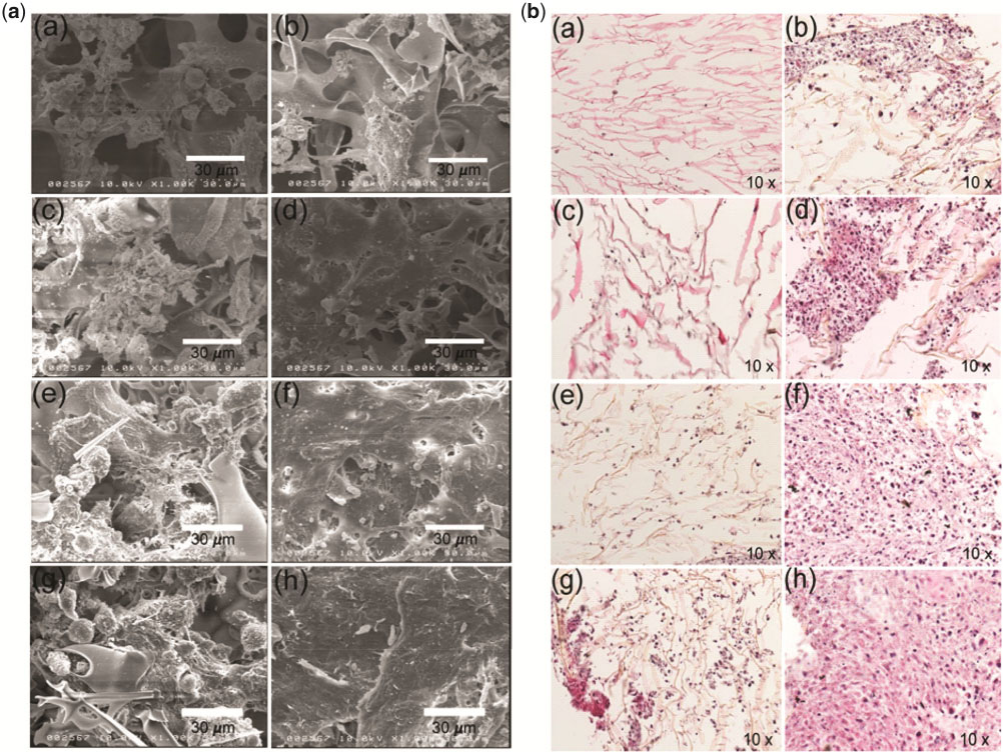
(**A**) SEM characterization of cell-cultured scaffold: images obtained from cell-cultured pristine chitosan scaffold fabricated (a, c, e and g) and RGD functionalized chitosan scaffold (b, d, f and h) of which a and b obtained at day 7, c and d at day 14, e and f at day 21 and g and h at day 28 of post seeding. Scale bar 30 μm. (**B**) Histochemistry of hMSC-cultured scaffold: H&E stained images of histologic section obtained from cell-cultured pristine chitosan scaffold (a, c, e and g) and RGD functionalized chitosan scaffold (b, d, f and h) of which a and b obtained at day 7, c and d at day 14, e and f at day 21 and g and h at day 28 of post seeding. Images were obtained using 10× objectives

The performance of RGD functionalized chitosan surface was evaluated by analysing the morphology of hMSC cultivated chitosan scaffold with SEM. For this, hMSC were seeded on both RGD functionalized and pristine scaffolds maintained with identical condition for 4 weeks and images were obtained every week. SEM images from hMSC cultured in pristine scaffold (left column) and RGD functionalized chitosan scaffold (right column) are shown in Fig. 5a. The time-dependent increase in densities of cells was reported from both scaffold groups. However, a significant difference in surface covering with cells has been observed between RGD functionalized and non-functionalized scaffold. The pristine chitosan scaffold was poorly occupied with hMSC leaving the unoccupied space even at the 28 days of post seeding, which indicates their slow growth tendency. Whereas, the RGD functionalized chitosan scaffold was occupied faster, most of the surface was occupied at 14-day post seeding (Fig. 5Ad) and no space remained for attaching newly proliferated cells at 21 and 28 day (Fig. 5Af and h) indicating their rapid growth tendency of hMSC on RGD functionalized chitosan scaffold surface. These morphological features of SEM images are consistent with the growth curve obtained from cell counting (Fig. 3e). These features of cell proliferation are obvious in the artificial surfaces, because of the continuous proliferative nature of hMSC and others, continuous cell lines [41]. These features of cell growth and proliferations on scaffold surfaces only, however majority of cell adhesion inside the scaffold need to be uncovered. This supports the previous findings in literature, showing that the cell migration and proliferation in porous scaffolds is highly influenced by the pore structure and their interconnectivity as they influence the availability of nutrients, growth factors and interchanging cell-to-cell signals [3]. Hence, variations in hMSC migration and migrations inside of the RGD functionalized and pristine scaffold needs to be investigated.

### Histochemistry of hMSC-cultured scaffold

To uncover cell migration and proliferation inside scaffolds, hMSC were cultured and maintained in pristine and RGD functionalized chitosan scaffolds for 4 weeks. Histochemistry of hMSC-cultured scaffold was performed every week using hematoxylin (H, nuclear stain) and eosin (E, cytoplasmic stain). For this, the cell-cultured scaffolds were fixed with 4% paraformaldehyde solution and subsequently embedded in paraffin wax. The chitosan scaffold in paraffin block was subjected to microtomic section for achieving thin (5 µm) section [31]. Then, sections were stained with H&E for visualizing nucleus (blue) and cytoplasm (brick red), respectively (Fig. 5B). The H&E staining sections clearly showed the distribution of hMSC along pores of the cultured scaffolds. The hMSC migration and proliferation efficiencies of 3D chitosan scaffold with RGD in comparison with scaffold without RGD was examined by analysing the stained transversely sectioned scaffold. The images obtained from hMSC proliferation in pristine scaffold are plotted in Fig. 5B (left panel) and hMSC proliferation in RGD functionalized scaffold are plotted in Fig. 5B (left panel). The images were taken at every week, that is, at the end of 7 day (Fig. 5Ba and b), 14 day (Fig. 5Bc and d), 21 day (Fig. 5Be and f) and 28 day (Fig. 5Bg and h). The results reveal that hMSC readily proliferated and distributed throughout the chitosan scaffold in presence of RGD. The hMSC was found to proliferate slowly and unevenly distribute in chitosan scaffold without RGD. This feature of growth tendency was noted throughout the experiment period. Intact, in absence of RGD modification, most of the scaffold surface remained uncovered with cell (Fig. 5B left panel). On other hand, in the RGD-modified scaffolds, very little space remained uncovered at 7 day (Fig. 5Bb) and 14 day (Fig. 5Bd), but entire surface was occupied at 21 day (Fig. 5Bf) and become a tissue-like structure at 28-day (Fig. 5Bh). This is interesting for regenerative medicine, interfaces for neural implant and may open new avenues for synthetic skin for artificial prosthetics and robotics [42–45]. RGD functionalized surfaces have been reported to involve receptor-mediated binding with integrin receptors on the cell membrane and enhancing FAK pathway-mediated cell adhesion, migration and proliferation [22, 23]. Therefore, this enhanced proliferation rates of hMSC is most likely due to the RGD functionalization of chitosan scaffold, which may have interacted with the integrin receptors on the cell surface membrane more easily [20]. These results clearly show that the RGD functionalized chitosan properly represents the adhesion ligands for cellular receptors, resulting in strong cell adhesion and increased proliferation [15, 16, 20].

### Mechanical stability of the cell-cultured scaffolds

**Figure 6:**
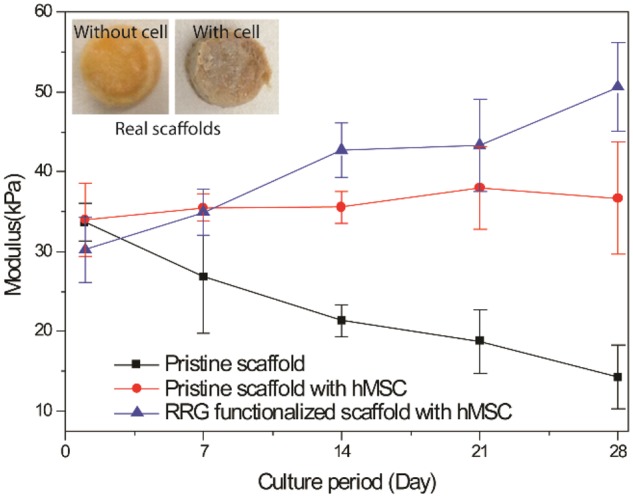
Effect of hMSC proliferation on compressive behavior of porous chitosan scaffold. All values are expressed as mean ± standard deviation from three individual samples. The significant difference (*P* < 0.05) was compared with pristine scaffold without hMSC

The RGD functionalized chitosan scaffold was further evaluated to know if there is any mechanical variation before and after cell adhesion, proliferation and migration. For this, both the pristine and RGD functionalized chitosan scaffolds were seeded with hMSC and maintained in Dulbecco's modified eagle medium (DMEM) providing standard culture condition for 4 weeks. The non-seeded pristine scaffolds were also maintained with similar condition in DMEM as a control. Mechanical testing was performed at weekly interval and the results are given in Fig. 6. The results reveal that compressive modulus for RGD functionalized chitosan scaffold started with lower modulus at day 1, increased after hMSC seeding and maintained the increased moduli throughout the experiment as compared with the pristine and control group. The little decrease in moduli at the beginning might be due to the several washing steps during the RGD functionalization [28]. The compressive moduli from pristine scaffold with hMSC maintained similar levels throughout the experiment period, whereas a gradual decrease in compressive moduli with experimental period was noticed for the control scaffold. The degradation behavior of chitosan material might be responsible for this decrease in compressive moduli [11–13]. The gradually increased compressive moduli of RGD functionalized scaffold might be due to the rapid proliferation and migration of hMSC throughout the scaffold [9]. Whereas, poor growth and slow migration in pristine scaffold were just enough to compensate the degradability and maintain the constant moduli throughout the experiment. This mechanical stability of hMSC-cultured RGD functionalized chitosan scaffold is essential for developing engineered tissue implant and their biomedical applications.

## Conclusions

Chitosan scaffolds with two different mechanical properties were fabricated at two different freezing TM (−80°C and −30°C) with the phase separation followed by lyophilization process. The samples fabricated at lower freezing TM (−80°C) produced scaffold with uniformity in pore structure with inter connected pores and higher degree of mechanical stability compared with the scaffold fabricated at higher freezing TM (−30°C). The scaffolds functionalized with RGD peptide required overcoming challenges such as self-assembly of cysteine-terminated RGD on thiolated chitosan scaffold. The performance of the presented scaffolds was evaluated with adhesion and proliferation of hMSCs. The adhesion and proliferation was observed to increase (150 and 300%, respectively) significantly with RGD functionalized scaffold as compared with RGD non-functionalized pristine scaffold. The enhanced cell adhesion and proliferation is due to increased binding sites for integrin receptor on the cell surface, which contributes to the activation of FA pathway. This phenomenon of RGD-integrin activation was confirmed by increased expressions of p-paxillin, p-FAK and p-ERK from cell grown on the RGD functionalized scaffold. The RGD enhanced cell proliferation was also verified by SEM images and histochemistry of the hMSC-cultured chitosan scaffold. Finally, mechanical stability of the RGD functionalized chitosan scaffold was determined by the mechanical testing. These superior features possible with RGD functionalized scaffold are essential to support 3D growth of cells and achieve tissue architecture. The tissue-like structures obtained with RGD functionalized scaffold is interesting as this may open new avenues for regenerative medicine and interfaces for neural.
